# SU16f inhibits fibrotic scar formation and facilitates axon regeneration and locomotor function recovery after spinal cord injury by blocking the PDGFRβ pathway

**DOI:** 10.1186/s12974-022-02449-3

**Published:** 2022-04-16

**Authors:** Ziyu Li, Shuisheng Yu, Yanchang Liu, Xuyang Hu, Yiteng Li, Zhaoming Xiao, Yihao Chen, Dasheng Tian, Xinzhong Xu, Li Cheng, Meige Zheng, Juehua Jing

**Affiliations:** Department of Orthopaedics, The Second Hospital of Anhui Medical University, 678 Furong Road, Hefei, 230601 Anhui China

**Keywords:** Spinal cord injury, Fibrotic scar, PDGFRβ, Platelet-derived growth factor, SU16f

## Abstract

**Background:**

Excessively deposited fibrotic scar after spinal cord injury (SCI) inhibits axon regeneration. It has been reported that platelet-derived growth factor receptor beta (PDGFRβ), as a marker of fibrotic scar-forming fibroblasts, can only be activated by platelet-derived growth factor (PDGF) B or PDGFD. However, whether the activation of the PDGFRβ pathway can mediate fibrotic scar formation after SCI remains unclear.

**Methods:**

A spinal cord compression injury mouse model was used. In situ injection of exogenous PDGFB or PDGFD in the spinal cord was used to specifically activate the PDGFRβ pathway in the uninjured spinal cord, while intrathecal injection of SU16f was used to specifically block the PDGFRβ pathway in the uninjured or injured spinal cord. Immunofluorescence staining was performed to explore the distributions and cell sources of PDGFB and PDGFD, and to evaluate astrocytic scar, fibrotic scar, inflammatory cells and axon regeneration after SCI. Basso Mouse Scale (BMS) and footprint analysis were performed to evaluate locomotor function recovery after SCI.

**Results:**

We found that the expression of PDGFD and PDGFB increased successively after SCI, and PDGFB was mainly secreted by astrocytes, while PDGFD was mainly secreted by macrophages/microglia and fibroblasts. In addition, in situ injection of exogenous PDGFB or PDGFD can lead to fibrosis in the uninjured spinal cord, while this profibrotic effect could be specifically blocked by the PDGFRβ inhibitor SU16f. We then treated the mice after SCI with SU16f and found the reduction of fibrotic scar, the interruption of scar boundary and the inhibition of lesion and inflammation, which promoted axon regeneration and locomotor function recovery after SCI.

**Conclusions:**

Our study demonstrates that activation of PDGFRβ pathway can directly induce fibrotic scar formation, and specific blocking of this pathway would contribute to the treatment of SCI.

## Background

The inhibitory microenvironment composed of an inflammatory response in the acute phase and scar tissue formation in the chronic phase is considered to be the main reason that hinders axon regeneration after spinal cord injury (SCI) [1, 2]. Because previous studies mostly focused on astrocytic scar formed by astrocytes after SCI, fibrotic scar formed by fibroblasts is less well understood [2, 3]. Following SCI, perivascular fibroblasts leave the blood vessel, proliferate, migrate and deposit fibrous extracellular matrix (ECM), including fibronectin, laminin and collagen, finally forming fibrotic scar inside the astrocytic scar, which hinders axon regeneration [4–7]. Moderate inhibition of fibrotic scar formation contributes to axon regeneration and locomotor function recovery, indicating that the adverse effects of excessively deposited fibrotic scar are greater than the beneficial effects after SCI [6, 8]. These results reveal that the role of fibrotic scar in the targeted therapy of SCI is of great significance and should be given more attention. However, the molecular mechanism of fibrotic scar formation after SCI is unclear.

Platelet-derived growth factors (PDGFs) are a cysteine-knot-type growth factor family composed of four polypeptide chains A, B, C, and D [9]. These growth factors activate intracellular signalling by binding to platelet-derived growth factor receptor (PDGFR) α or PDGFRβ, while PDGFRβ can only be activated by PDGFB and PDGFD and plays an important role in cell proliferation, differentiation and migration [10–12]. A large number of studies have shown that the PDGF/PDGFR pathway is a critical functional mediator of neurodegenerative diseases, including Alzheimer’s disease (AD), Parkinson’s disease (PD) and amyotrophic lateral sclerosis (ALS), while relatively little is known about it in SCI [12]. It has been reported that PDGFRβ, as a marker of perivascular fibroblasts, is expressed in almost all scar-forming fibroblasts [5, 6, 13], while whether the PDGFRβ pathway is involved in fibrotic scar formation after SCI is still lacking direct evidence. Our previous in vitro study showed that PDGFB can regulate the migration of fibrotic scar-forming cells model PDGFRβ^+^ pericytes/fibroblasts, which can be aborted by the PDGFRβ inhibitor SU16f [14]. Nevertheless, the cellular location and function of the PDGF/PDGFRβ pathway after SCI need to be further explored in vivo.

In this study, our results showed that the expression of PDGFD occurred earlier than that of PDGFB after SCI, and PDGFB was mainly secreted by astrocytes, while PDGFD was mainly secreted by macrophages/microglia and fibroblasts. Intrathecal injection of the PDGFRβ inhibitor SU16f blocked the fibrosis induced by exogenous PDGFB or PDGFD in the uninjured spinal cord. In addition, SU16f blockade of the PDGFRβ pathway resulted in the reduction and interruption of fibrotic scar and the resolution of lesion and inflammation, thereby facilitating axon regeneration and locomotor function recovery after SCI. These results indicate that the PDGFRβ pathway is essential for fibrotic scar formation after SCI and is expected to be a therapeutic target for SCI.

## Materials and methods

### Animals and spinal cord compression injury model

All experiments involving animals were approved by the Ethics Committee of Anhui Medical University (Approval No. LLSC20160052). Eight-week-old C57BL/6 mice were acquired from the Animal Experiment Center of Anhui Medical University and were housed in an environment with controlled temperature and humidity and a 12:12 h light:dark cycle. The animals were randomly grouped and kept in standardized cages, where water and food were readily available.

The establishment of the spinal cord compression injury model has been described in detail in our previous study [14]. In brief, after satisfactory anaesthesia with isoflurane (induction 4%, maintenance 2%), the mid-thoracic level (T10) spinal cord was carefully exposed and compressed with calibrated Dumont #5 forceps (11252-20, Fine Science Tools, Germany) for 5 s. The postoperative mice received anti-infection treatment and auxiliary urination nursing twice a day.

### In situ injection of PDGFB or PDGFD

The object of in situ injection of PDGFB was the uninjured spinal cord of mice. The T10 spinal cord was exposed according to the established method of the spinal cord injury model, and then the mouse was fixed on the stereotaxic device. The insertion site of the microinjection needle (7634-01 and 7803-05, Hamilton, Switzerland) was 0.3 mm lateral to the midline and 0.8 mm deep to the dorsal surface of the mouse spinal cord [15]. Two microlitres of 100 ng/μl recombinant human PDGFB (HZ-1308, Proteintech, China) dissolved in 10 mM HOAc containing 0.1% bovine serum albumin (BSA) or PDGFD (1159-SB/CF, R&D Systems, United States) dissolved in 4 mM HCl containing 0.1% BSA was injected into the uninjured spinal cord at 0.5 μl/min using a stereotaxic injector (KDS LEGATO 130, RWD, China). The control mice received 2 μl of 10 mM HOAc containing 0.1% BSA or 4 mM HCl containing 0.1% BSA. All mice were sacrificed at 7 days after injection.

### Intrathecal injection of SU16f

The needle insertion site was located in the dorsal midpoint of the lumbar 5–6 intervertebral space as previously reported [16]. It was confirmed that the needle was successfully inserted into the intradural space by observing an evident sudden tail flick. Ten microlitres of 3 mM SU16f dissolved in 0.1 M phosphate buffered saline (PBS) containing 3% DMSO (3304, R&D Systems, United States) was injected daily at 1 μl/4 s using a microinjection needle (1701, Hamilton, Switzerland). For the mice without SCI, SU16f was preinjected the day before the injection of PDGFB or PDGFD and then injected daily for 7 consecutive days from the day of PDGFB or PDGFD injection. For the mice with SCI, SU16f was injected from 3 day post-injury (dpi) until sacrifice. The control mice received 10 μl of PBS containing 3% DMSO.

### Intraperitoneal injection of Bromodeoxyuridine (BrdU)

To label proliferating fibroblasts, mice received intraperitoneal injection of 50 mg/kg body weight BrdU (BS916, Biosharp, China) daily for 1–6 dpi. All mice were sacrificed at 7 dpi.

### Tissue preparation and immunofluorescent staining

After cardiac perfusion with 0.1 M PBS (Servicebio, China) followed by 4% paraformaldehyde (PFA, Servicebio, China), the 0.5 mm segment of spinal cord tissue containing the injured core was placed in 4% PFA and postfixed for 5 h. The tissue was then placed in a 30% sucrose solution and dehydrated at 4 °C for 24 h until the tissue sank to the bottom. Finally, the tissue was cut into 18 μm-thick serial sagittal or coronal sections using a cryostat (NX50, Thermo Fisher Scientific, United States). The sections encompassing the lesion core or injection site were used.

For BrdU staining, the sections were pretreated with 2 N hydrochloric acid (HCl, GEMIC, China) at 37 °C for 30 min followed by 0.1 M borate buffer (KGR0101, KeyGEN BioTECH, China) at room temperature for 10 min and were subjected to an immunofluorescence staining protocol. The sections were blocked in 10% donkey serum containing 0.3% Triton X-100 (SL050 and T8200, Solarbio, China) at room temperature for 1 h, followed by incubation with primary antibodies at 4 °C overnight. The primary antibodies included goat anti-PDGFRβ (5 μg/ml, AF1042-SP, R&D Systems, United States), goat anti-CD31 (1:100, AF3628, R&D Systems, United States), goat anti-5-hydroxytryptamine (5-HT) (1:5000, 20079, Immunostar, United States), rabbit anti-PDGFB (1:100, NBP1-58279, Novus, United States), rabbit anti-PDGFD (1:100, 40–2100, Thermo Fisher Scientific, United States), rabbit anti-fibronectin (1:100, 15613-1-AP, Proteintech, China), rabbit anti-laminin (1:100, 23498-1-AP, Proteintech, China), rabbit anti-neurofilament (NF) (1:500, ab207176, Abcam, United States), rat anti-GFAP (1:400, 13-0300, Thermo Fisher Scientific, United States), rat anti-CD68 (1:400, MCA1957, Bio-Rad, United States), rat anti-BrdU (1:200, ab6326, Abcam, United States) and rat anti-Ki67 (1:100, 14-5698-80, Thermo Fisher Scientific, United States). Subsequently, the sections were incubated with appropriate secondary antibodies at room temperature for 1 h, including donkey anti-goat Alexa Fluor 488, donkey anti-goat Alexa Fluor 555, donkey anti-goat Alexa Fluor 647, donkey anti-rabbit Alexa Fluor 555, donkey anti-rat Alexa Fluor 488 and donkey anti-rat Alexa Fluor 555 (1:500, A-11055, A-21432, A-21447, A-31572, A-21208, A48270, Thermo Fisher Scientific, United States). Finally, the sections were stained with DAPI (C1005, Beyotime Biotechnology, China) to label the nuclei. The negative control sections were incubated with secondary antibody alone.

### Image acquisition and quantitative analysis

Representative images of the sections were acquired using a Zeiss LSM 900 confocal microscope system and a Zeiss Axio Scope A1 fluorescence microscope. Staining colocalization was determined using ZEN 3.3 software to examine each of the ten one-micron Z-stack slices. Image processing was performed using ImageJ version 2.0 (NIH, United States).

All quantitative analyses were performed in a blind fashion. To quantify GFAP^+^, CD68^+^, CD31^+^, PDGFRβ^+^, PDGFB^+^ and PDGFD^+^ cells, 100 μm square grids were generated over the injured site [17]. Every 6th square was quantified, and only DAPI^+^ cells were counted. One section encompassing the lesion core in each sample was used for counting, with 5 samples per group.

To evaluate the area of fibrotic scar, the immunoreactivities of PDGFRβ, fibronectin and laminin were normalized to the area of the spinal cord segment spanning the injured core in a 4 × image [17]. Similarly, the GFAP^−^ area and CD68^+^ area was normalized to the area of the spinal cord segment spanning the injured core in a 4 × image. To evaluate axon regeneration, the immunoreactivity of 5-HT was normalized to the area of the spinal cord segment spanning the injured core in a 10 × image, and the number of NF^+^ axons longer than 1 μm in the GFAP^−^ region was counted and normalized to the area of the GFAP^−^ region. For each sample, sections spanning the injured core and two adjacent sections spaced 180 μm apart were quantified, and the results from each section were averaged, with 5 samples per group.

To evaluate the proliferation of fibroblasts, BrdU^+^ PDGFRβ^+^ or Ki67^+^ PDGFRβ^+^ cells were counted on 40 × images spanning the injured core. The average of three random 40 × images was used as the final result of each sample, with 5 samples per group.

### Behavioural assessments

The Basso Mouse Scale (BMS) is widely used to evaluate locomotor function recovery after SCI in mice [18]. In this study, BMS was performed in an open field according to the protocol developed by Basso and colleagues [19]. All mice received BMS to confirm normal locomotor function before SCI and received BMS to confirm the success of the SCI model after surgery. Each mouse was assessed by two experienced examiners at 3, 7, 14, 21 and 28 dpi, and the average value was finally obtained, with 8 animals per group.

Footprint analysis was used to further evaluate locomotor function recovery at 28 dpi and was performed according to previous reports [20]. The mice without SCI received footprint analysis were included in the uninjured group. The front paws were dipped in green dyes, and the hind paws were dipped in red dyes. The stride length was determined by the distance from the beginning to the end of the hind paw in a step. The stride width was determined by the distance from the outermost toe of the left paw to the outermost toe of the right paw. The paw rotation was determined by the angle between the midline axis of the body and the axis of the hind paw. All assessments were performed in three consecutive gait cycles on each side and averaged, with 8 animals per group.

All behavioural assessments were performed in a blind fashion.

### Statistical analysis

The data are presented as the mean ± standard error of the mean (SEM), and individual data points are plotted in the figures. The statistical methods used are presented in the figure legends. Multiple comparisons were analysed with one-way or two-way analysis of variance (ANOVA) with a post hoc Tukey–Kramer test, and comparisons between two groups were performed using Student’s *t* test. Data analysis and chart production were performed using GraphPad Prism 8.0 (GraphPad, United States), and a value of *p* < 0.05 was considered statistically significant.

## Results

### Different spatiotemporal distributions of PDGFB and PDGFD after SCI

**Fig. 1:**
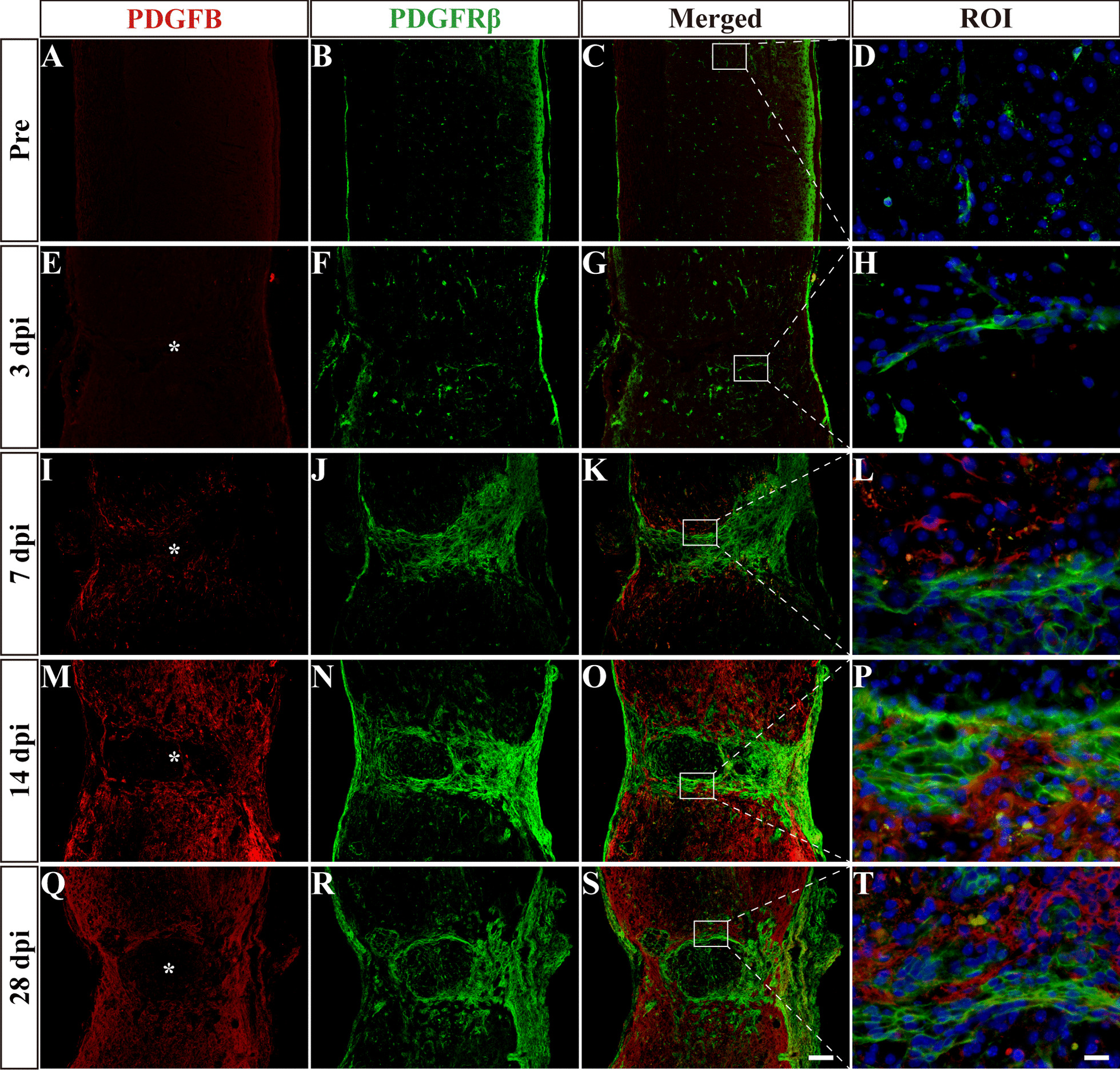
Spatiotemporal distribution of PDGFB and PDGFRβ after SCI. Immunofluorescence staining of PDGFB (red), PDGFRβ (green) and nuclei (blue) in sagittal sections before SCI and at 3, 7, 14 and 28 dpi. The region of interest (ROI) represents the boxed region on the left. Asterisks indicate the injured core. Scale bars: 200 μm in **S** and 20 μm in **T**

**Fig. 2:**
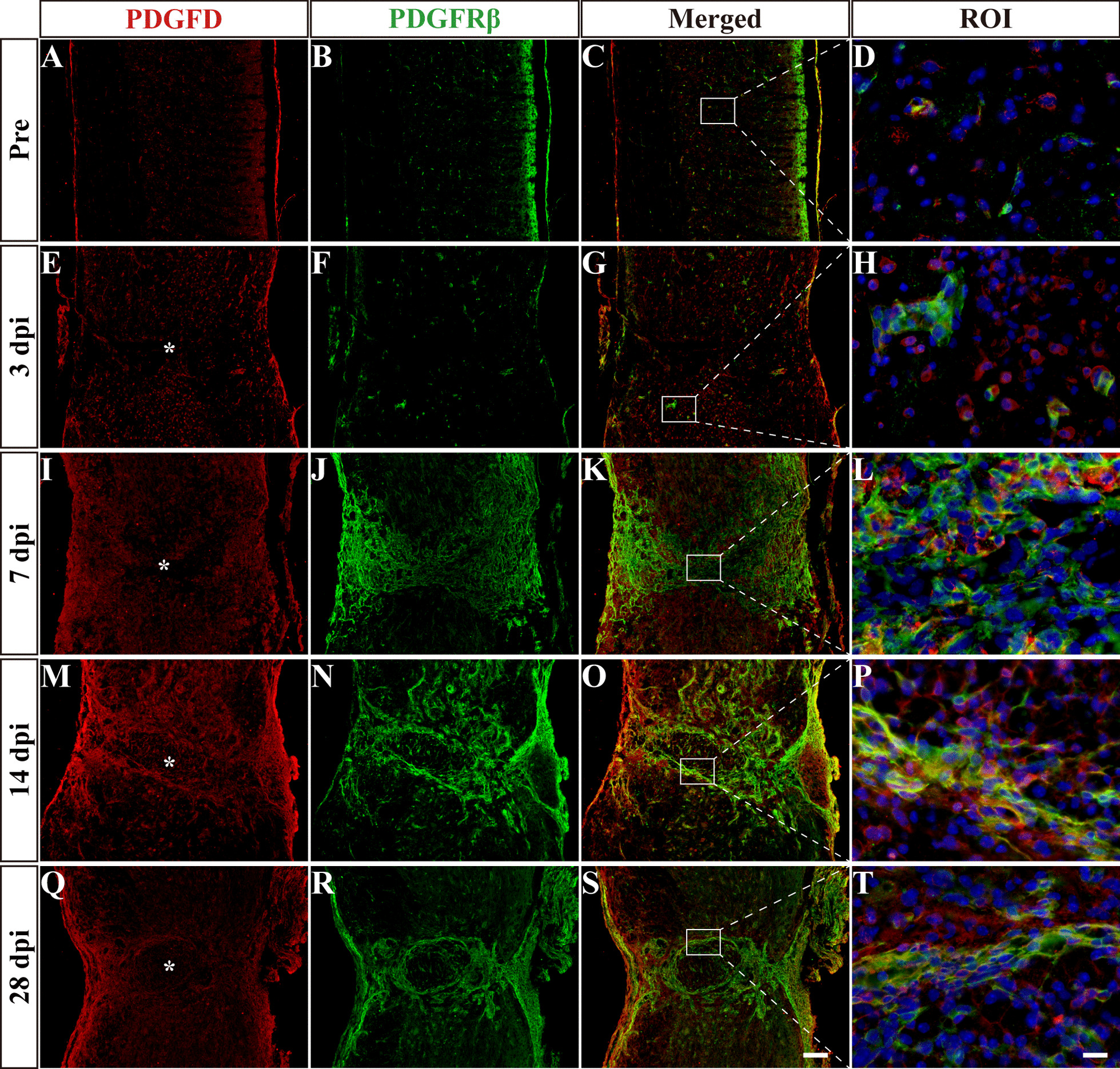
Spatiotemporal distribution of PDGFD and PDGFRβ after SCI. Immunofluorescence staining of PDGFD (red), PDGFRβ (green) and nuclei (blue) in sagittal sections before SCI and at 3, 7, 14 and 28 dpi. The region of interest (ROI) represents the boxed region on the left. Asterisks indicate the injured core. Scale bars: 200 μm in **S** and 20 μm in **T**

After SCI, PDGFRβ is expressed in all fibrotic scar-forming fibroblasts [13], and PDGFRβ can only be activated by PDGFB and PDGFD to participate in a variety of biological processes, including peripheral organ fibrosis [10–12]. We speculated that PDGFRβ is involved in the formation of fibrotic scar after SCI. Therefore, immunofluorescent staining was performed to confirm the spatiotemporal distribution of PDGFB, PDGFD and PDGFRβ after SCI. The results showed that the PDGFRβ^+^ fibroblasts increased significantly and aggregated gradually to the injured site at 3 to 7 dpi, while a contiguous fibrotic scar boundary formed to corral the injured core at 14 to 28 dpi (Figs. 1, 2), which was consistent with previous studies [4–6]. Meanwhile, PDGFB and PDGFD were widely expressed and distributed adjacent to PDGFRβ after SCI until 28 dpi (Figs. 1, 2). These results suggest that there may be an interaction between the ligand PDGFB or PDGFD and the receptor PDGFRβ, which may be involved in fibrotic scar formation after SCI. Interestingly, PDGFB was significantly expressed around the lesion epicentre from 7 dpi (Fig. 1I–T), while PDGFD was expressed earlier from 3 dpi (Fig. 2E–H) and preferentially distributed at the lesion epicentre at 14–28 dpi (Fig. 2M–T). These results suggest that PDGFB and PDGFD may have different cell sources after SCI.

### PDGFB is mainly secreted by astrocytes, while PDGFD is mainly secreted by macrophages/microglia and fibroblasts after SCI

**Fig. 3:**
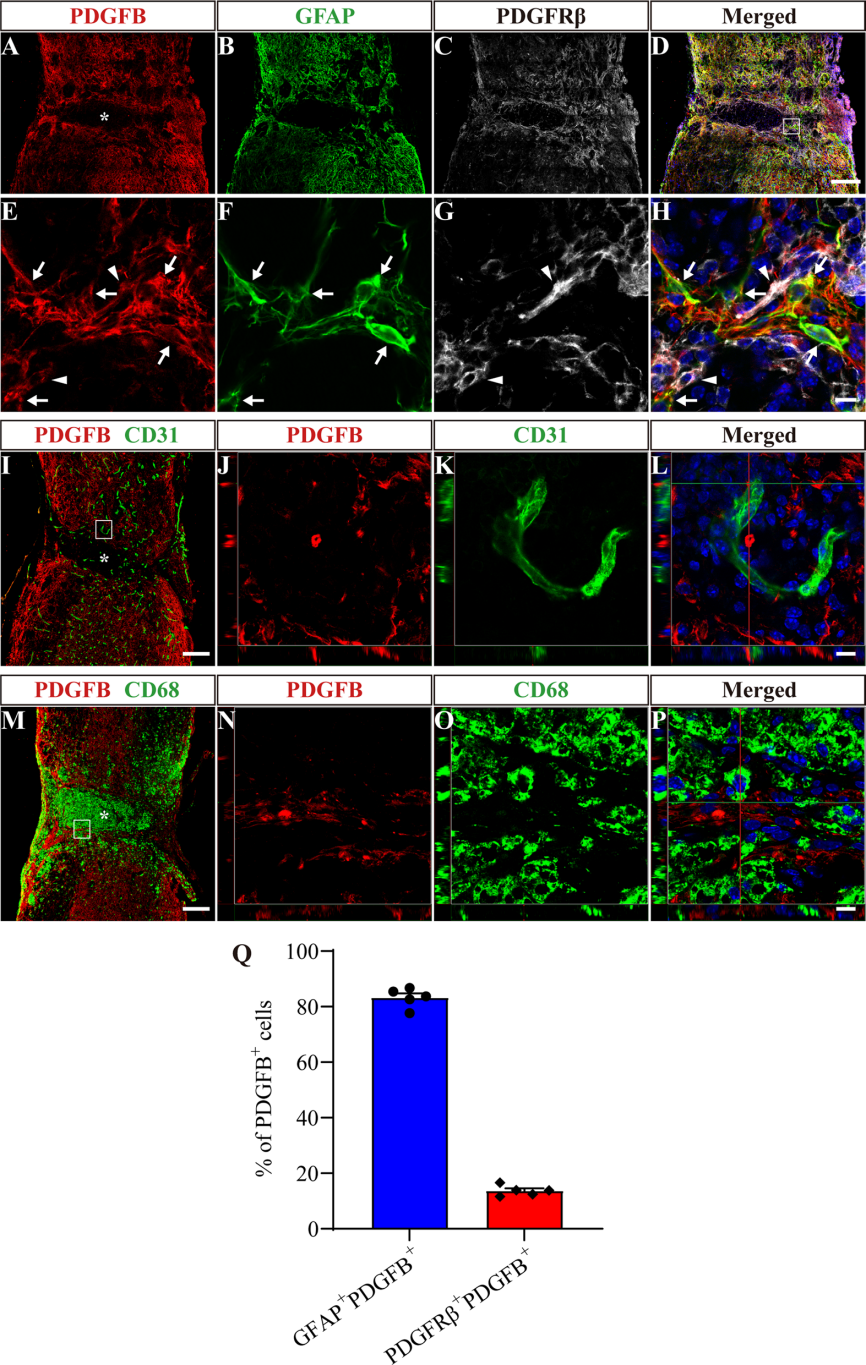
PDGFB is mainly secreted by astrocytes after SCI. **A**–**D** Immunofluorescence staining of PDGFB (red), GFAP (green) and PDGFRβ (white) in sagittal sections at 14 dpi. **E**–**H** Higher magnification images of the boxed region in **D**. Arrows indicate GFAP^+^PDGFB^+^ cells. Arrowheads indicate PDGFRβ^+^PDGFB^+^ cells. **I**–**L** Immunofluorescence staining of PDGFB (red), CD31 (green) and nuclei (blue) in sagittal sections at 14 dpi. **J**–**L** represent boxed region in **I**. **M**–**P** Immunofluorescence staining of PDGFB (red), CD68 (green) and nuclei (blue) in sagittal sections at 14 dpi. **N**–**P** represent boxed region in **M**. **Q** Quantification of the proportion of GFAP^+^PDGFB^+^ cells or PDGFRβ^+^PDGFB^+^ cells in PDGFB^+^ cells at 14 dpi. Asterisks indicate the injured core. Scale bars: 200 μm in **D, I and M** and 10 μm in **H, L and P**. *n* = 5 animals per group

To preliminarily explore the cell sources of PDGFB and PDGFD, we detected their costaining with the main cell components of the injury site, including macrophages/microglia, fibroblasts, astrocytes and vascular endothelial cells. GFAP was used to label astrocytes, CD31 was used to label vascular endothelial cells, and CD68 was used to label macrophages/microglia. The staining results showed substantial colocalization between PDGFB and GFAP^+^ astrocytes or PDGFRβ^+^ fibroblasts at 14 dpi (Fig. 3A–H). GFAP^+^PDGFB^+^ cells and PDGFRβ^+^PDGFB^+^ cells accounted for 83.26 ± 1.56% and 13.69 ± 0.85% of PDGFB^+^ cells, respectively (Fig. 3Q). There was no significant colocalization between PDGFB and CD68^+^ macrophages/microglia or CD31^+^ vascular endothelial cells (Fig. 3I–P). Furthermore, GFAP^+^PDGFB^+^ cells and PDGFRβ^+^PDGFB^+^ cells were adjacent to each other at the edge of the injured core (Fig. 3H). These results indicate that PDGFB is mainly secreted by astrocytes after SCI.

**Fig. 4:**
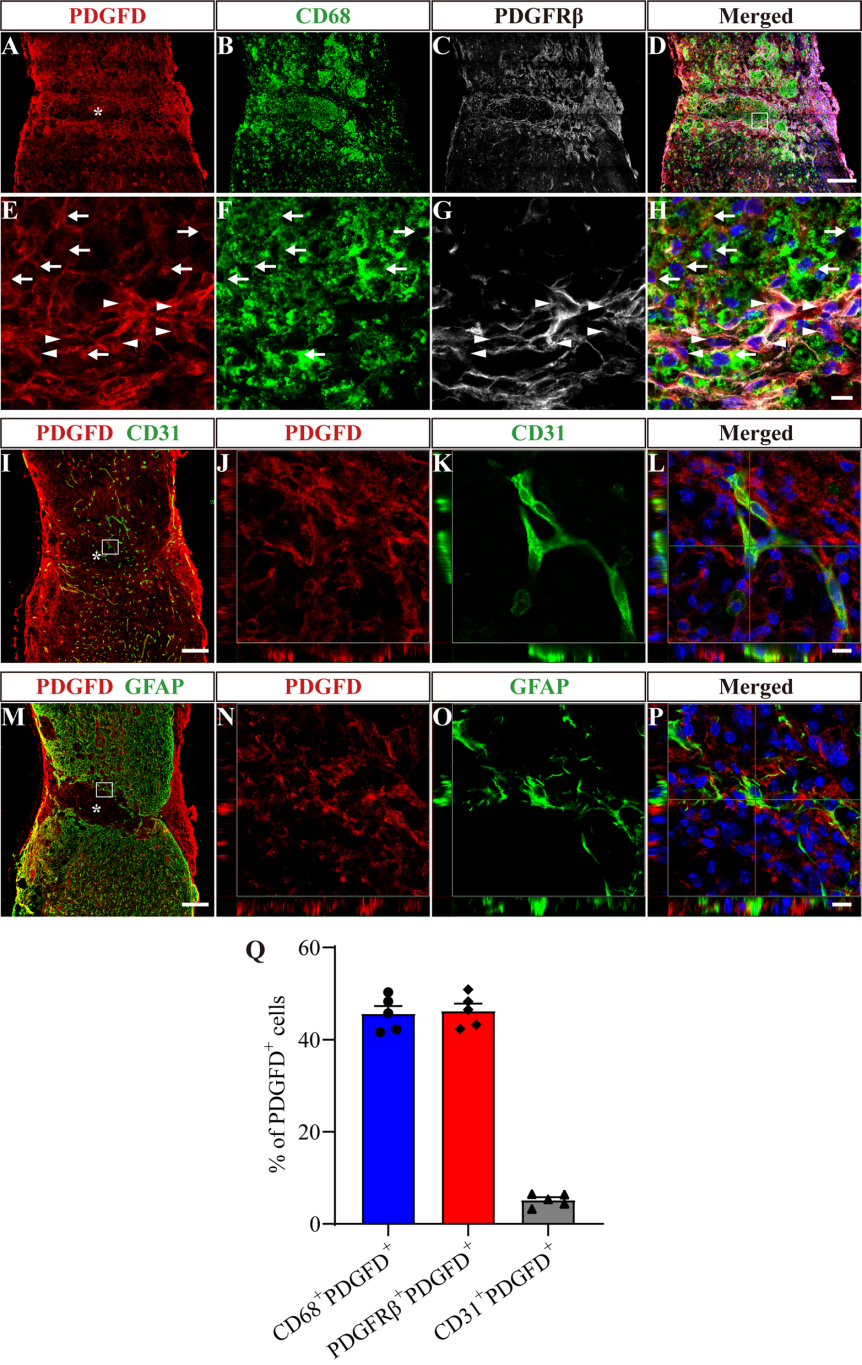
PDGFD is mainly secreted by macrophages/microglia and fibroblasts after SCI. **A**–**D** Immunofluorescence staining of PDGFD (red), CD68 (green) and PDGFRβ (white) in sagittal sections at 14 dpi. **E**–**H** Higher magnification images of the boxed region in **D**. Arrows indicate CD68^+^PDGFD^+^ cells. Arrowheads indicate PDGFRβ^+^PDGFD^+^ cells. **I**–**L** Immunofluorescence staining of PDGFD (red), CD31 (green) and nuclei (blue) in sagittal sections at 14 dpi. **J**–**L** represents boxed region in **I**. **M**–**P** Immunofluorescence staining of PDGFD (red), GFAP (green) and nuclei (blue) in sagittal sections at 14 dpi. **N**–**P** represents boxed region in **M**. **Q** Quantification of the proportion of CD68^+^PDGFD^+^ cells, PDGFRβ^+^PDGFD^+^ cells or CD31^+^PDGFD^+^ cells in PDGFD^+^ cells at 14 dpi. Asterisks indicate the injured core. Scale bars: 200 μm in **D, I and M** and 10 μm in **H, L and P**. *n* = 5 animals per group

PDGFD mainly colocalized with CD68^+^ macrophages/microglia or PDGFRβ^+^ fibroblasts at 14 dpi (Fig. 4A–H), and CD68^+^PDGFD^+^ cells accounted for 45.63 ± 1.68%, while PDGFRβ^+^PDGFD^+^ cells accounted for 46.23 ± 1.59% of PDGFD^+^ cells (Fig. 4Q). There was slight colocalization between PDGFD and CD31^+^ vascular endothelial cells and no colocalization between PDGFD and GFAP^+^ astrocytes (Fig. 4I–P). CD31^+^PDGFD^+^ cells accounted for 5.18 ± 0.61% of PDGFD^+^ cells (Fig. 4Q). PDGFRβ^+^PDGFD^+^ cells were in close contact with CD68^+^PDGFD^+^ cells at the injured core (Fig. 4H). These results indicate that PDGFD is mainly secreted by macrophages/microglia and fibroblasts after SCI. Overall, our results reveal that PDGFD is expressed earlier by macrophages/microglia and fibroblasts gathered in the injured core, while the expression of PDGFB is delayed and mainly secreted by astrocytes surrounding the injured core.

### In situ injection of PDGFB or PDGFD induces fibrosis in the uninjured spinal cord

**Fig. 5:**
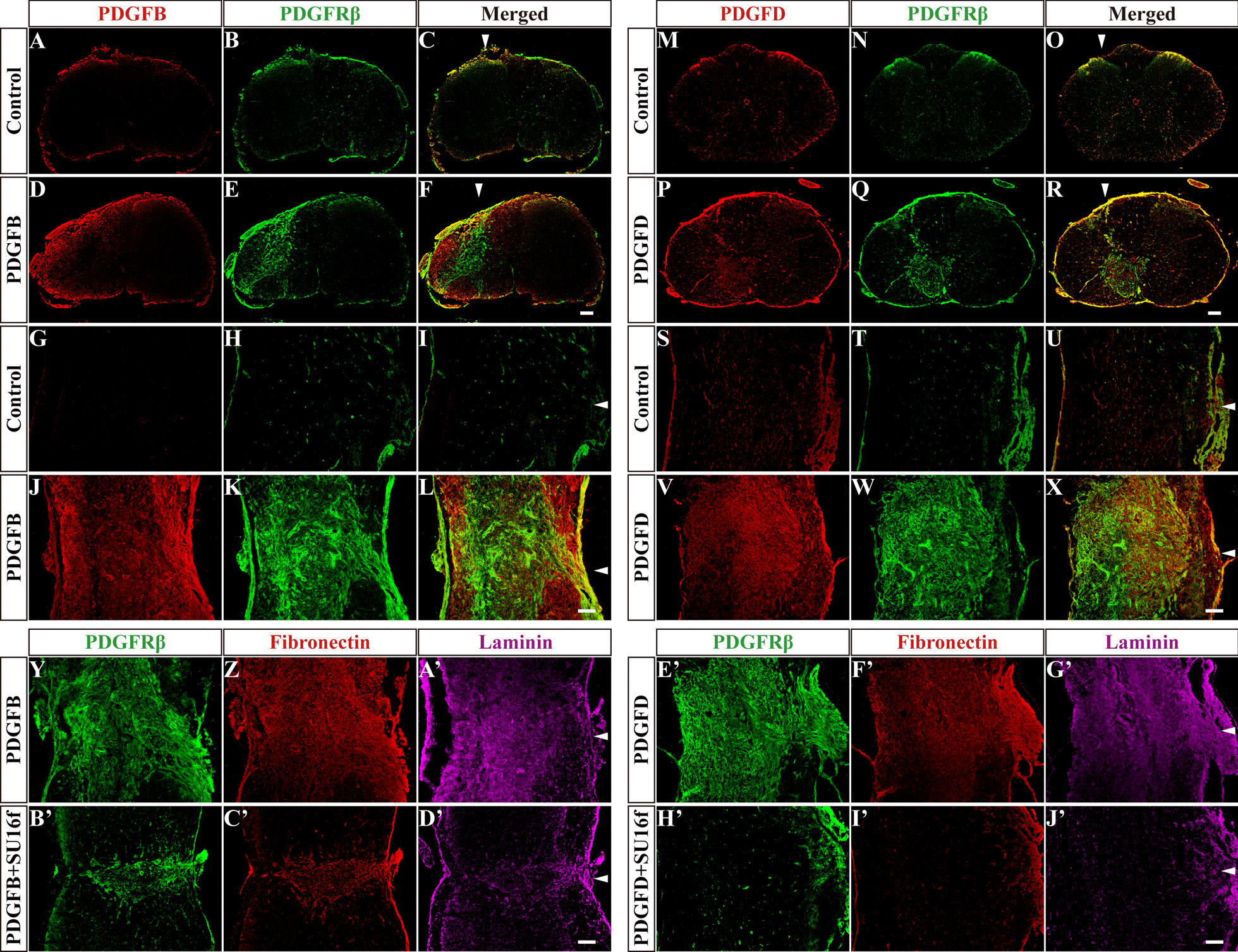
In situ injection of PDGFB or PDGFD induces fibrosis in uninjured spinal cord. **A**–**L** Immunofluorescence staining of PDGFB (red) and PDGFRβ (green) in sagittal and coronal sections of the Control and PDGFB groups at 7 days after injection. **M**–**X** Immunofluorescence staining of PDGFD (red) and PDGFRβ (green) in sagittal and coronal sections of the Control and PDGFD groups at 7 days after injection. **Y**–**D’** Immunofluorescence staining of PDGFRβ (green), fibronectin (red) and laminin (violet) in sagittal sections of the PDGFB and PDGFB + SU16f groups at 7 days after injection. **E’**–**J’** Immunofluorescence staining of PDGFRβ (green), fibronectin (red) and laminin (violet) in sagittal sections of the PDGFD and PDGFD + SU16f groups at 7 days after injection. Arrowheads indicate the sites of in situ injection. Scale bars: 200 μm

To directly investigate the effect of the PDGFRβ pathway, a single factor, on fibrotic scar formation after SCI, we injected exogenous PDGFB or PDGFD into the uninjured spinal cord to activate the PDGFRβ pathway. Immunofluorescence staining was used to detect PDGFRβ, fibronectin and laminin to observe the changes in fibroblasts and fibrous ECM. The results of the control group showed that the injection itself did not lead to PDGFB or PDGFD expression and fibroblast aggregation (Fig. 5A–C, M–O, G–I and S–U). Compared with the control group, the injection of PDGFB or PDGFD alone induced a large number of PDGFRβ^+^ fibroblasts to accumulate in the uninjured spinal cord (Fig. 5A–X) and triggered an excessive accumulation of fibrous ECM, including fibronectin and laminin (Fig. 5Y–A’, E’–G’), leading to fibrosis at 7 days after injection. Notably, similar to the distribution after SCI (Figs. 1M–P, 2M–P), the injected PDGFB was mainly located at the outer edge of the fibrosis core (Fig. 5D–F, J–L), while PDGFD was mainly located at the fibrosis core in the uninjured spinal cord (Fig. 5P–R, V–X). Therefore, our results indicate that the activation of the PDGFRβ pathway by PDGFB or PDGFD can directly induce fibrosis in the uninjured spinal cord.

To further verify the specific role of the PDGFRβ pathway in fibrotic scar formation, intrathecal injection of SU16f was used to block the activation of PDGFRβ in the uninjured spinal cord that received the injection of PDGFB or PDGFD. SU16f is a potent and highly selective PDGFRβ inhibitor that displays > 14-fold, > 229-fold and > 10,000-fold selectivity over VEGFR2, FGFR1 and EGFR, respectively, and SU16f has been used to specifically block PDGFRβ [21–24]. In our study, SU16f was preinjected the day before the injection of PDGFB or PDGFD and then injected daily for 7 consecutive days from the day of PDGFB or PDGFD injection. Our results showed that the fibrosis, as determined by PDGFRβ^+^, fibronectin^+^ and laminin^+^ staining, was significantly reduced in the uninjured spinal cord of the mice that received the combined injection of SU16f and PDGFB or PDGFD (Fig. 5B’–D’, H’–J’) compared with that of the mice that received the injection of PDGFB or PDGFD alone (Fig. 5Y–A’, E’–G’). These results indicate that the PDGFRβ pathway inhibitor SU16f can block exogenous PDGFB- or PDGFD-induced fibrosis in the uninjured spinal cord. Interestingly, SU16f completely blocked PDGFD-induced fibrosis, but only partially blocked PDGFB-induced fibrosis. These results suggest that PDGFB and PDGFD may be involved in different phases of fibrotic scar formation, and we emphasize that the process and mechanism are worthy of in-depth study. Overall, excluding the influence of the complex microenvironment after SCI, we used this method to more specifically confirm that the PDGFRβ pathway is a sufficient factor for fibrosis.

### Intrathecal injection of the PDGFRβ blocker SU16f inhibits fibroblasts proliferation and fibrotic scar formation after SCI

**Fig. 6:**
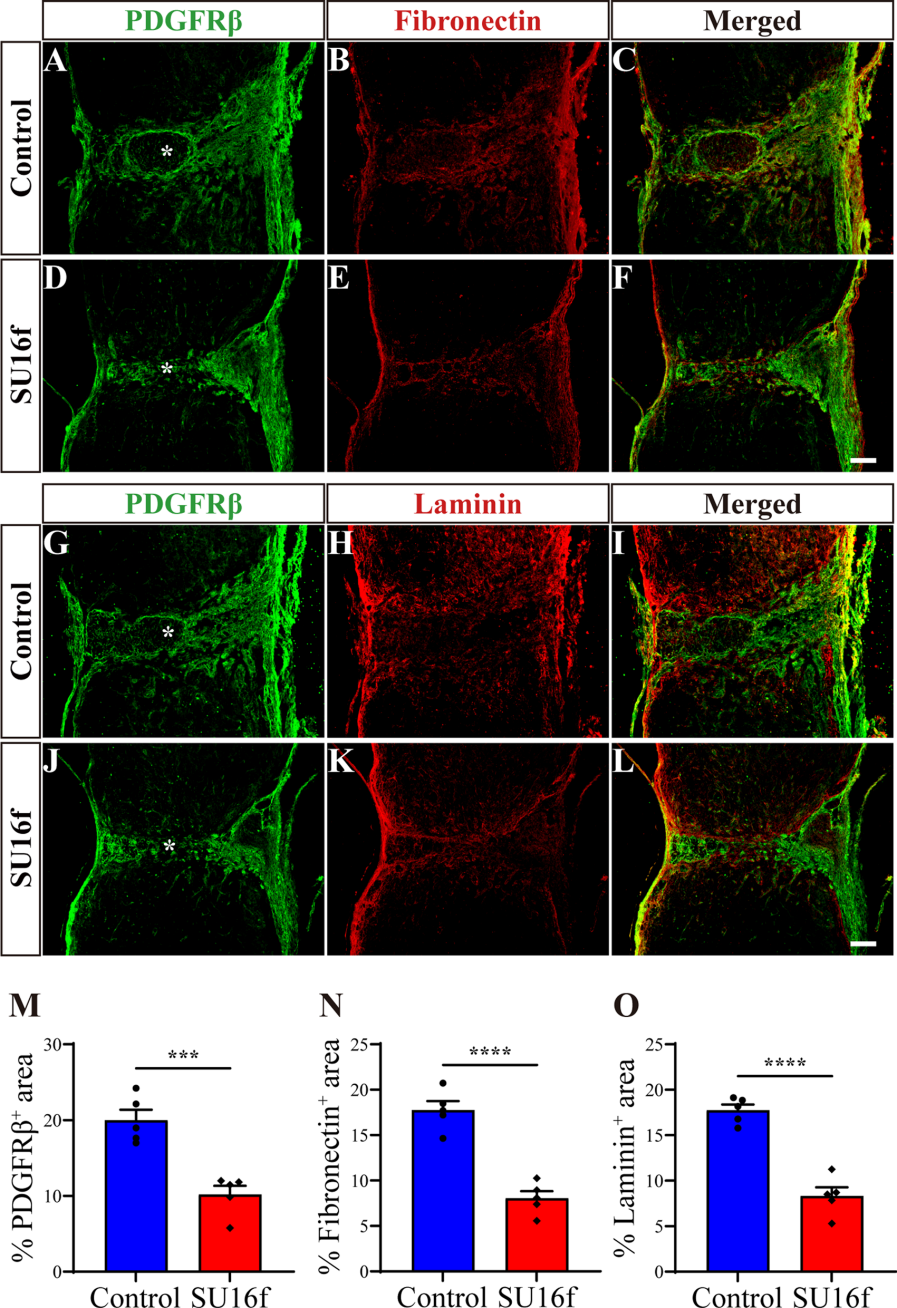
Intrathecal injection of SU16f reduces fibrotic scar after SCI. **A**–**F** Immunofluorescence staining of fibronectin (red) and PDGFRβ (green) in sagittal sections of the Control and SU16f groups at 28 dpi. **G**–**L** Immunofluorescence staining of laminin (red) and PDGFRβ (green) in sagittal sections of the Control and SU16f groups at 28 dpi. **M**–**O** Quantification of the percentage of PDGFRβ^+^ area, fibronectin^+^ area or laminin^+^ area in the area of the spinal cord segment spanning the injured core at 28 dpi. Asterisks indicate the injured core. Scale bars: 200 μm. ***P < 0.001 and ****P < 0.0001 by Student’s *t* test, *n* = 5 animals per group

To further confirm the role of the PDGFRβ pathway in regulating fibrotic scar formation after SCI, intrathecal injection of SU16f was used to treat the mice with SCI. The injured spinal cord is in the period of apoptosis and necrosis at 3 dpi, from which fibroblasts begin to proliferate and aggregate in the injured site [5]. Therefore, we performed daily intrathecal injection of SU16f from 3 dpi to block the PDGFRβ pathway (Fig. 10A). Immunofluorescence staining showed that the fibrotic scar, manifested as the PDGFRβ^+^, fibronectin^+^ and laminin^+^ areas, was significantly reduced at 28 dpi after the intrathecal injection of SU16f compared with the control group (Fig. 6). These results indicate that SU16f blockade of the PDGFRβ pathway can inhibit the formation of fibrotic scar after SCI, resulting in a reduction in fibrotic scar area.

**Fig. 7:**
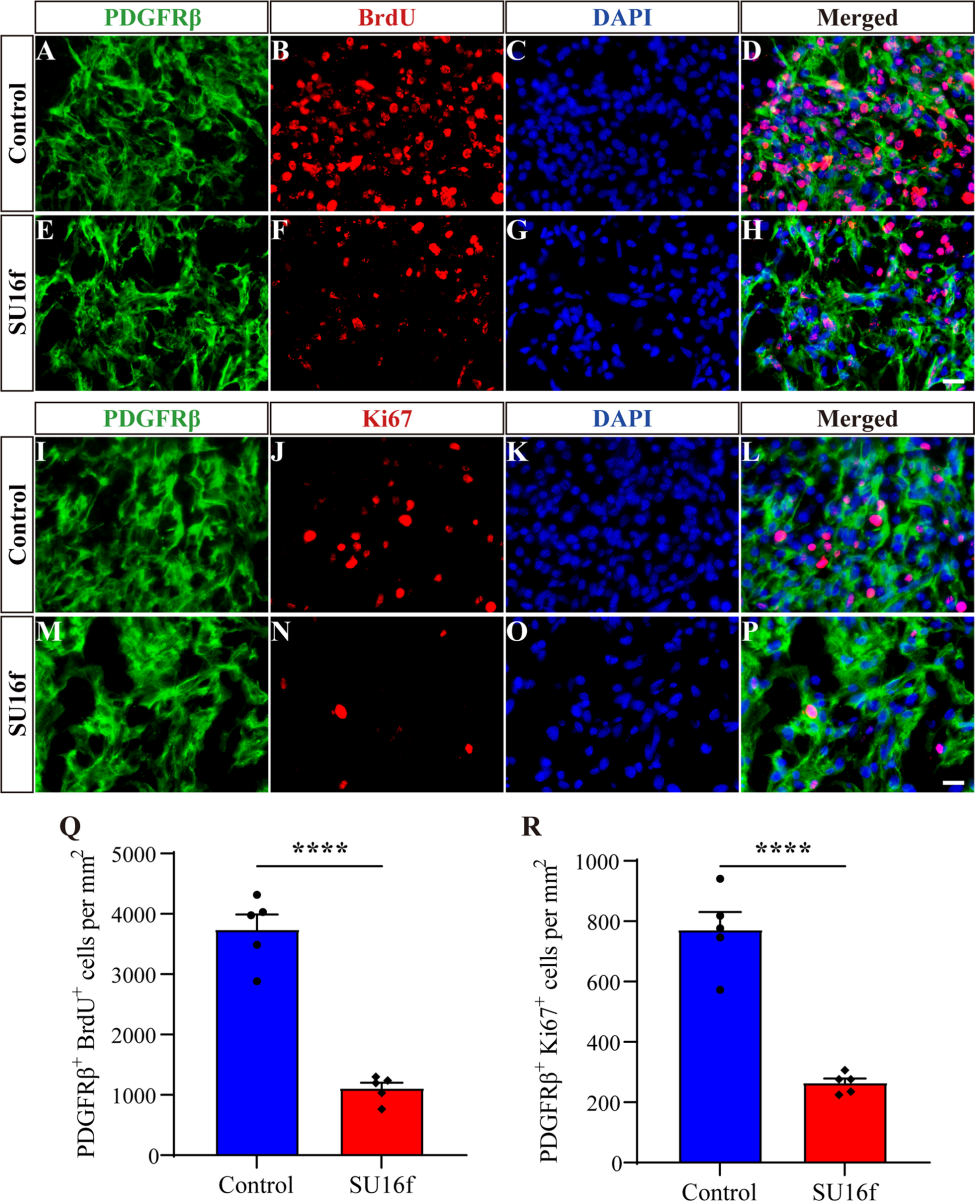
Intrathecal injection of SU16f inhibits fibroblasts proliferation after SCI. **A**–**H** Immunofluorescence staining of BrdU (red), PDGFRβ (green) and nuclei (blue) in sagittal sections of the Control and SU16f groups at 7 dpi. **I**–**P** Immunofluorescence staining of Ki67 (red), PDGFRβ (green) and nuclei (blue) in sagittal sections of the Control and SU16f groups at 7 dpi. **Q**–**R** Quantification of the density of BrdU^+^PDGFRβ^+^ cells **Q** or Ki67^+^PDGFRβ^+^ cells **R** at 7 dpi. Scale bars: 20 μm. ****P < 0.0001 by Student’s *t* test, *n* = 5 animals per group

It has been reported that the number of fibroblasts reaches its peak at 7 dpi, which is mainly caused by the proliferation of fibroblasts inherent in the spinal cord, suggesting that fibroblasts proliferation is an important process of fibrotic scar formation after SCI [4]. Therefore, immunofluorescence staining detecting BrdU and Ki67 was used to evaluate the effect of SU16f on the proliferation of fibroblasts at 7 dpi. The results showed that the density of BrdU^+^PDGFRβ^+^ cells (Fig. 7A–H, Q) and Ki67^+^PDGFRβ^+^ cells (Fig. 7I–P, R) in the SU16f group was significantly lower than that of the control group at 7 dpi. These results suggest that SU16f blockade of the PDGFRβ pathway can inhibit the proliferation of fibroblasts after SCI, which may contribute to the reduction in fibrotic scar.

### Intrathecal injection of SU16f breaks the fibrotic/astrocytic scar boundary, shrinks the lesion and inhibits inflammation after SCI

**Fig. 8:**
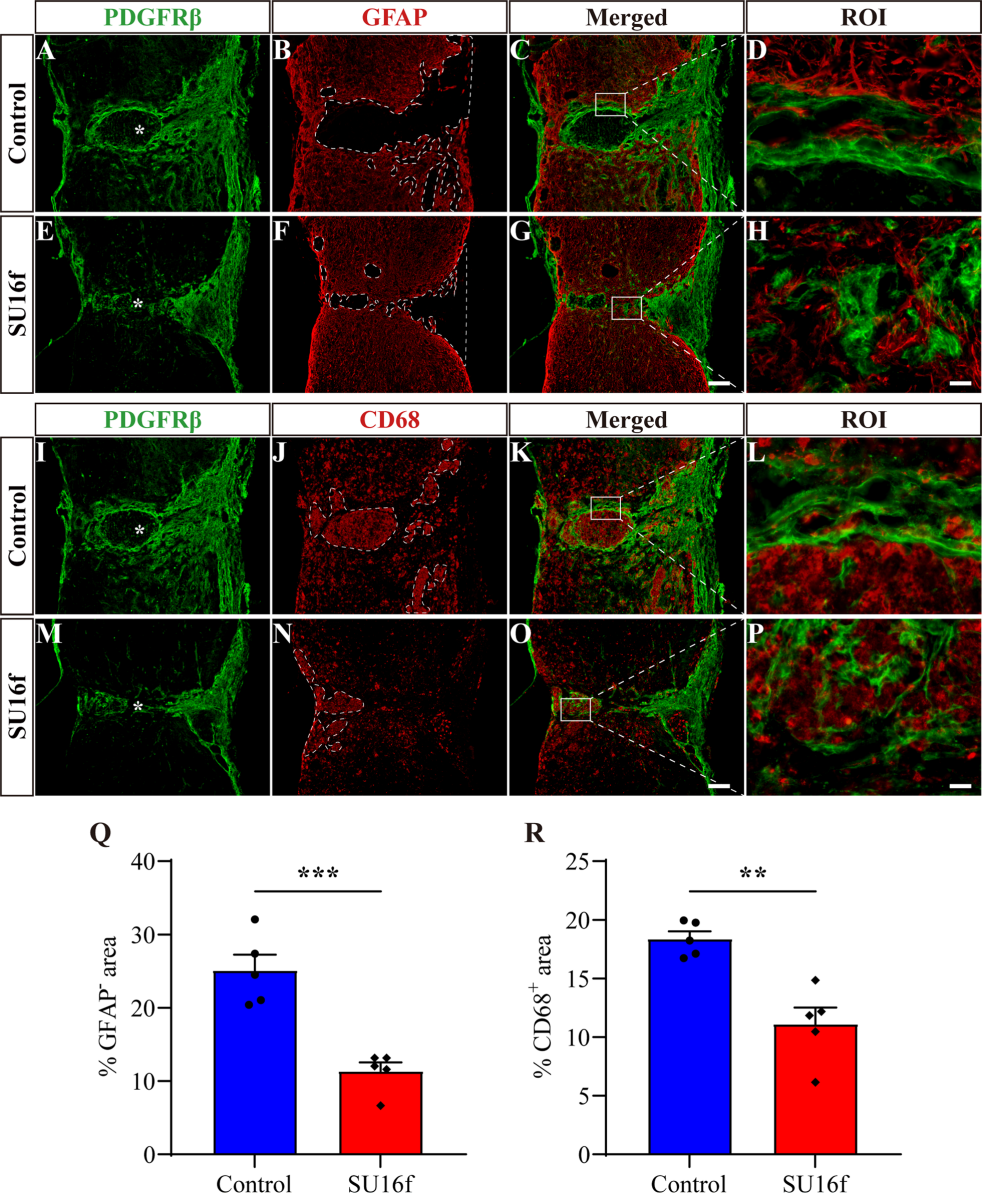
Intrathecal injection of SU16f breaks the scar boundary, inhibits the lesion and inflammation after SCI. **A**–**H** Immunofluorescence staining of GFAP (red) and PDGFRβ (green) in sagittal sections of the Control and SU16f groups at 28 dpi. The region of interest (ROI) represents the boxed region on the left and shows the fibrotic/astrocytic scar boundary. **I**–**P** Immunofluorescence staining of CD68 (red) and PDGFRβ (green) in sagittal sections of the Control and SU16f groups at 28 dpi. ROI represents boxed region in the left. **Q**–**R** Quantification of the percentage of GFAP^−^ area **Q** or CD68^+^ area **R** in the area of the spinal cord segment spanning the injured core at 28 dpi. Asterisks indicate the injured core. Scale bars: 200 μm in **G** and **O** and 20 μm in **H** and **P**. **P < 0.01 and ***P < 0.001 by Student’s *t* test, *n* = 5 animals per group

Following SCI, astrocytic scar and fibrotic scar form a dense and contiguous boundary surrounding the injured core, which is one of the important reasons for the failure of axon regeneration [1, 6, 17]. Therefore, we further explored the effect of SU16f blockade of PDGFRβ on the fibrotic/astrocytic scar boundary, lesion size and inflammation after SCI. GFAP was used to label astrocytic scar and CD68 was used to label inflammatory cells after SCI. The results showed that compared with the control group, the GFAP^−^ area indicated that the lesion size was significantly reduced at 28 dpi after the intrathecal injection of SU16f (Fig. 8A–H, Q). The contiguous boundary of the fibrotic/astrocytic scar was oriented parallel to the injured core at 28 dpi in the control group (Fig. 8D, L). However, after intrathecal injection of SU16f, the astrocytic/fibrotic scar was disordered and was oriented perpendicular to the injured core at 28 dpi (Fig. 8H, P). Therefore, our results indicate that SU16f blockade of the PDGFRβ pathway breaks the fibrotic/astrocytic scar boundary and shrinks the lesion after SCI. Breaking of the scar barrier may facilitate the passage of regenerated axons through the injured core, which has been assessed below.

It has been reported that fibrotic scar corrals inflammatory cells to the injured core after SCI, contributing to limiting inflammation [6, 17]. However, compared with the control group, SU16f inhibition of fibrotic scar formation led to a significant decrease in the CD68^+^ inflammatory cell area at 28 dpi (Fig. 8I–P, R). These results suggest that moderate inhibition of fibrotic scar by SU16f blockade of the PDGFRβ pathway contributes to the reduction in inflammation in the chronic phase of SCI.

### Intrathecal injection of SU16f promotes axon regeneration and locomotor function recovery after SCI

**Fig. 9:**
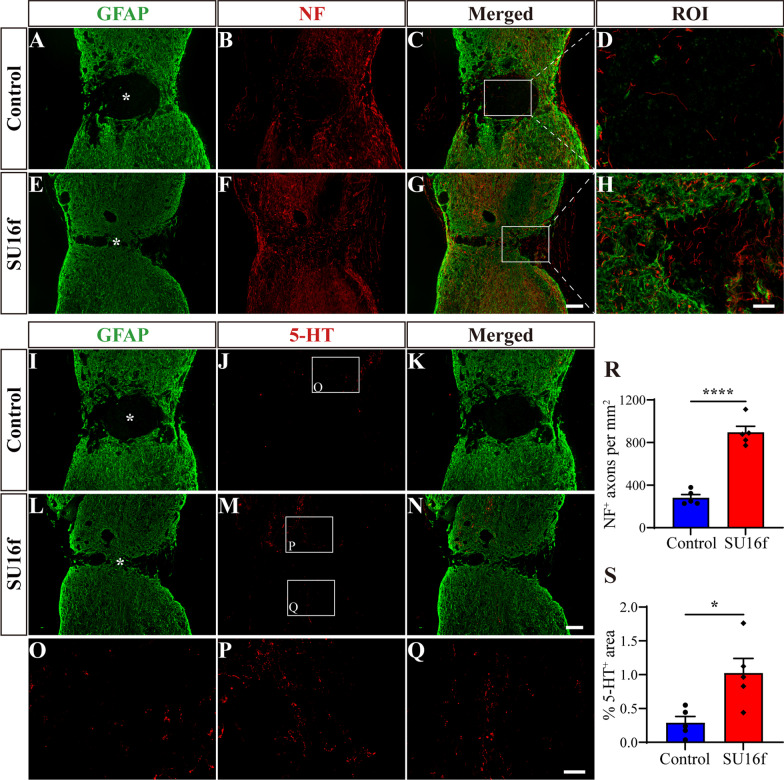
Intrathecal injection of SU16f promotes axon regeneration after SCI. **A**–**H** Immunofluorescence staining of NF (red) and GFAP (green) in sagittal sections of the Control and SU16f groups at 28 dpi. The region of interest (ROI) represents the boxed region on the left. **I**–**N** Immunofluorescence staining of 5-HT (red) and GFAP (green) in sagittal sections of the Control and SU16f groups at 28 dpi. **O**–**Q** Higher magnification images of the boxed region in **J** and **M**. **R** Quantification of the density of NF^+^ axons in the GFAP^−^ area at 28 dpi. **S** Quantification of the percentage of 5-HT^+^ area in the area of the spinal cord segment spanning the injured core at 28 dpi. Asterisk indicates the injured core. Scale bars: 200 μm in **G** and **N**, 20 μm in **H** and 50 μm in **Q**. *P < 0.05 and ****P < 0.0001 by Student’s *t* test, *n* = 5 animals per group

To further confirm whether the PDGFRβ pathway can be used as a therapeutic target for SCI and the effect of SU16f on axon regeneration after SCI, immunofluorescence staining was used to assess the regeneration of NF^+^ or 5-HT^+^ axons. The GFAP^−^ area was used to distinguish the injured core. The results showed that compared with the control group, the NF^+^ axon density of the injured core in the SU16f group increased significantly after SCI (Fig. 9A–H, R). In addition, SU16f significantly increased the area of 5-HT^+^ axons of the injured site after SCI (Fig. 9I–P, S). Three out of 5 mice in the SU16f group presented regenerated 5-HT^+^ axons that passed through the injured core to the caudal side after SCI, which was not observed in the control group (Fig. 9J, M and Q). These results indicate that SU16f blockade of the PDGFRβ pathway contributes to axon regeneration after SCI, which may be caused by reduced fibrotic scar and inflammation (Fig. 8).

**Fig. 10:**
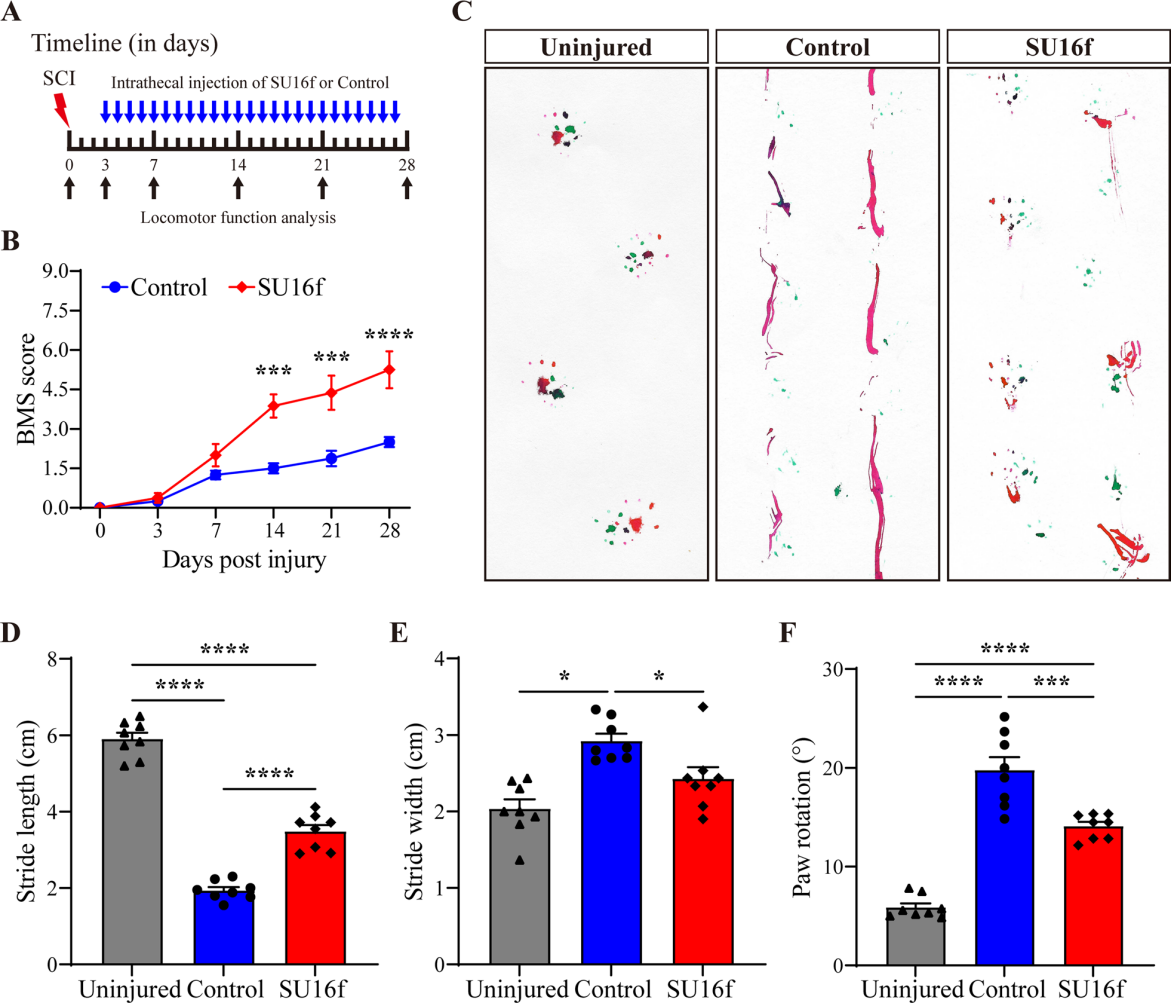
Intrathecal injection of SU16f promotes locomotor function recovery after SCI. **A** Timeline of the intrathecal injection and the behavioural assessment. **B** Locomotor function was evaluated by BMS at 0, 3, 7, 14, 21 and 28 dpi. ***P < 0.001 and ****P < 0.0001 vs. Control group by two-way ANOVA, *n* = 8 animals per group. **C** Representative images of footprint analysis in the Uninjured, Control and SU16f groups at 28 dpi. The front paws are shown in green dyes, and the hind paws are shown in red dyes. **D**–**F** Quantification of the stride length, stride width and paw rotation at 28 dpi. *P < 0.05, ***P < 0.001 and ****P < 0.0001 by one-way ANOVA, *n* = 8 animals per group

Furthermore, BMS score and footprint analysis were used to analyse the recovery of locomotor function after SCI. Compared with the mice in the control group, the mice injected with SU16f obtained better hind limb locomotor function at 14, 21 and 28 dpi, corresponding to a higher BMS score (Fig. 10B). In addition, footprint analysis further revealed that the mice in the SU16f group obtained better locomotor function at 28 dpi (Fig. 10C), including longer stride length, shorter stride width and smaller paw rotation, than the mice in the control group (Fig. 10D–F). Although the mice in the SU16f group obtained better treatment effects, their locomotor function did not return to the level of the uninjured group (Fig. 10C–F). Overall, our results indicate that blocking PDGFRβ with SU16f contributes to the recovery of locomotor function after SCI.

## Discussion

In this study, we found that the expression of PDGFD occurred earlier than that of PDGFB after SCI, and PDGFB was mainly secreted by astrocytes, while PDGFD was mainly secreted by macrophages/microglia and fibroblasts. Moreover, in situ injection of exogenous PDGFB or PDGFD can lead to fibrosis in the uninjured spinal cord, while SU16f blockade of the PDGFRβ pathway reduced the fibrotic scar area, interrupted the fibrotic/astrocytic scar boundary, shrunk the lesion and inhibited inflammation, promoting axon regeneration and locomotor function recovery after SCI. Therefore, the PDGFRβ pathway is expected to be a therapeutic target after SCI.

SCI is a devastating trauma and causes sensory and locomotor dysfunction in patients, and there is currently a lack of effective clinical treatments [25, 26]. Therefore, it is of great significance to explore the pathological changes and molecular mechanisms of SCI, so as to provide new ideas for treatment. Scars, as one of the critical factors hindering axon regeneration after SCI, mainly include fibrotic scar formed by fibroblasts and astrocytic scar formed by astrocytes [1, 5]. Although the deposition of chondroitin sulphate proteoglycans (CSPGs) by astrocytes leads to the failure of axon regeneration after SCI [27, 28], the secretion of axon-growth-supporting molecules by astrocytes is required for axon regeneration and astrocytic scar has the beneficial effect of limiting inflammation [29, 30]. Meanwhile, recent studies have shown that inhibition of astrocytic scar formation cannot promote axon regeneration, while astrocytic scar formation aids rather than prevents axon regeneration [29, 31], suggesting that the beneficial effects of astrocytic scar in SCI are greater than the adverse effects. However, moderate inhibition of fibrotic scar formation can promote axon regeneration and functional recovery after SCI [6, 17], indicating that fibrotic scar is of great significance as a therapeutic target for SCI.

Following SCI, perivascular fibroblasts leave blood vessels, proliferate and migrate to the injured site at 3–7 dpi [4, 5]. At 7–14 dpi, fibroblasts deposit large amounts of fibrous ECM, including fibronectin, laminin and collagen, to form fibrotic scar that corrals macrophages in the injured core and is located on the inner side of astrocytic scar [7, 17, 18]. It has been accepted that fibrotic scar significantly hinders axon regeneration after SCI [8, 32–34]. The Jonas Frisén group used Glast–Rasless transgenic mice to specifically block the proliferation of fibroblasts after SCI, thereby establishing a fibrotic scar removal model [4, 6]. In addition, they revealed that complete elimination of fibrotic scar leads to the failure of injured site closure and the spread of inflammation after SCI, while a moderate reduction in fibrotic scar inhibits inflammation and promotes axon regeneration after SCI [4, 6], suggesting that fibrotic scar can be used as a therapeutic target after SCI. However, previous reports mainly used transgenic strategies or nonspecific target intervention strategies to inhibit fibrotic scar formation after SCI [6, 8, 32]. For instance, transforming growth factor beta (TGF-β) not only is a profibrotic factor but also participates in a variety of biological processes [35, 36]. Administration of 8-Br-cAMP, Taxol, epothilone B (epoB) or antagomir-21 has been successfully used to suppress fibrotic scar after SCI via inhibiting TGFβ pathway, while targeting TGFβ is not specific for regulating fibrotic scar [32–34, 37]. Therefore, a better understanding of the molecular mechanism of fibrotic scar formation after SCI could lead to the uncovering of specific molecular therapeutic targets, which is of major significance.

PDGFRβ is a transmembrane receptor tyrosine kinase composed of an intracellular tyrosine kinase domain and an extracellular ligand binding domain [9]. PDGFB or PDGFD binding to PDGFRβ monomers mediates dimerization of PDGFRβ monomers and then activates kinase activity to trigger intracellular signalling cascades, including Janus kinase (JAK), phospholipase C gamma (PLCγ) and phosphoinositide 3-kinase (PI3K), involved in cell proliferation, differentiation and migration [10, 11]. It has been reported that PDGFRβ is expressed in pericytes, astrocytes, NG2 cells and endothelial cells in brain injury models [38]. However, PDGFRβ is widely used to label pericytes and regulates the survival, proliferation and migration of pericytes in the brain, thereby participating in angiogenesis and the repair and maintenance of the blood–brain barrier (BBB) [12]. In the injured spinal cord, PDGFRβ is expressed on all fibrotic scar-forming fibroblasts, and fibroblasts account for up to 95% of PDGFRβ^+^ cells, indicating that PDGFRβ is specifically expressed in fibroblasts after SCI [7, 8, 13]. However, the spatiotemporal distribution of the ligands PDGFB and PDGFD and the effect of activation of the PDGFRβ pathway on fibroblasts forming fibrotic scar after SCI remain unclear. In this study, we found that PDGFB and PDGFD were highly expressed and distributed adjacent to PDGFRβ after SCI, suggesting that PDGFB or PDGFD may activate PDGFRβ to be involved in the formation of fibrotic scar after SCI. In addition, PDGFB began to be expressed in a large area from 7 dpi and was gradually distributed around the lesion epicentre, while PDGFD began to be expressed in a large area from 3 dpi and was gradually distributed at the lesion epicentre. These results indicate that PDGFD may be mainly involved in the recruitment and proliferation of fibroblasts in the early stage, while PDGFB may be mainly involved in the assembly and maturation of fibrotic scar in the late stage. The functional difference between PDGFB and PDGFD needs to be further studied, which is expected to provide a theoretical basis for sequential intervention of the PDGFRβ pathway after SCI.

Fibroblasts, astrocytes, vascular endothelial cells and macrophages/microglia are important cellular components at the injured site of SCI, and recent evidence has demonstrated extensive crosstalk among them [17, 18, 39, 40]. The inhibition of fibrotic scarring results in the attenuation of astrogliosis and the interruption of astrocytic scar boundary after SCI [6]. Besides, the inhibition of astrocytic scarring leads to the interruption of fibrotic scar boundary and the spread of inflammation after SCI [39, 41], and the depletion of macrophages or microglia in the injured core leads to the interruption of fibrotic scar boundary after SCI [17, 18, 42]. However, the molecular mechanism cues for the crosstalk among the cells remain largely elusive. Therefore, we further investigated the cell sources of PDGFB and PDGFD and focused the sources on macrophages, astrocytes, vascular endothelial cells or fibroblasts, which was expected to provide a basis for the crosstalk among the main cell components at the injured site. Our results showed that PDGFB was mainly secreted by astrocytes, while PDGFD was mainly secreted by macrophages/microglia and fibroblasts after SCI. The different sources of PDGFB and PDGFD indicate their different functions after SCI, while whether the PDGF/PDGFRβ pathway plays a role in the crosstalk among astrocytes, macrophages/microglia and fibroblasts needs to be further investigated.

To directly explore the effect of the PDGFRβ pathway, a single factor, on fibrotic scar formation, we injected exogenous PDGFB or PDGFD into the uninjured spinal cord instead of the injured spinal cord to avoid the influence of the complex microenvironment of SCI. Our results showed that both PDGFB and PDGFD can promote fibrosis in the uninjured spinal cord, and the profibrotic effect can be blocked by the PDGFRβ inhibitor SU16f. The results of FN- or LN-labelled fibrosis was consistent with those of PDGFRβ-labelled fibrosis. Therefore, our results were reliable and preliminarily confirmed that the activation of the PDGFRβ pathway is sufficient to induce fibrosis. Notably, SU16f completely blocked PDGFD-induced fibrosis but only partially blocked PDGFB-induced fibrosis in the uninjured spinal cord, suggesting that PDGFB and PDGFD may be involved in different phases of fibrotic scar formation. We emphasize that the process and mechanism are worthy of in-depth study. In addition, SU16f blockade of the PDGFRβ pathway was performed to further confirm the effect of the PDGFRβ pathway on fibrotic scar formation after SCI. The results showed that SU16f significantly inhibited the proliferation of fibroblasts and reduced fibrotic scar after SCI. Therefore, our results provide direct evidence that the PDGFRβ pathway mediates fibrotic scar formation after SCI, which can be blocked by SU16f inhibiting the proliferation of fibroblasts.

The dense contiguous fibrotic/astrocytic scar boundary is an important component of the inhibitory microenvironment after SCI [1]. The physical barrier of the scars directly prevents the regenerated axons from passing through the injured core, and the axon tips form retraction bulbs after contacting fibrotic scar, resulting in the failure of axon regeneration after SCI [6]. Therefore, the interruption of the contiguous scar boundary contributes to axon regeneration [6]. In our study, the results showed that SU16f blockade of the PDGFRβ pathway resulted in the interruption of the fibrotic/astrocytic scar boundary and the reduction of the lesion size after SCI, facilitating the regeneration of NF^+^ or 5-HT^+^ axons that passed through the injured core. Interestingly, our results showed that SU16f-induced reduction in fibrotic scar led to a smaller area of inflammatory cells at 28 dpi. The Jonas Frisén group used Glast–Rasless transgenic mice to completely eliminate fibrotic scar after SCI, leading to the spread of inflammatory cells at 14 dpi. However, a moderate reduction in fibrotic scar did not lead to the spread of inflammatory cells at 14 dpi but led to a reduction in inflammatory cells at 28 dpi [4, 6]. Therefore, our results are consistent with the results of the Jonas Frisén group, together indicating that moderate inhibition of fibrotic scar after SCI does not lead to the spread of inflammation in the early stage but inhibits the spread of inflammation in the late stage, which contributes to axon regeneration. Blood-derived macrophages migrate towards high concentrations of complement component C5a in the injured core after SCI, and C5a may be secreted by PDGFRβ^+^ fibroblasts [43], suggesting that C5a may be involved in fibroblasts corralling macrophages in the injured core. The effect of fibrotic scar changes on inflammatory response after SCI and its molecular mechanism need to be further investigated. Overall, our results further reveal that the adverse effects of excessively deposited fibrotic scar are greater than its beneficial effects in SCI and can be used as a therapeutic target after SCI. Finally, the results of BMS score and footprint analysis confirmed that SU16f blockade of the PDGFRβ pathway promotes locomotor function recovery in injured mice. Although fibrotic scar forms after SCI in both rats and mice [7], SCI in rats leads to cavity formation in the injured core, which is considered to resemble the pathological changes in patients with SCI in the clinic [44, 45]. Our findings should be further validated in rat models, which could contribute to a better understanding of fibrotic scar as a therapeutic target for SCI in clinic. Thus far, specific therapeutic targets for inhibiting fibrotic scar formation after SCI have rarely been reported, and the present study is expected to provide a novel idea.

## Conclusion

The present study reveals that PDGFD and PDGFB increase successively after SCI and can activate PDGFRβ^+^ fibroblasts. PDGFD is mainly secreted by macrophages/microglia and fibroblasts and distributed at the lesion epicentre, while PDGFB is mainly secreted by astrocytes and distributed around the lesion epicentre. Intrathecal injection of the PDGFRβ inhibitor SU16f blocked the fibrosis induced by exogenous PDGFB or PDGFD in the uninjured spinal cord. Furthermore, blocking the PDGFRβ pathway with SU16f reduces fibrotic scar, interrupts scar boundary and inhibits lesion and inflammation, promoting axon regeneration and locomotor function recovery after SCI. This study confirms that the PDGF/PDGFRβ pathway plays a critical role in fibrotic scar formation after SCI and is expected to be a specific target for the treatment of SCI.

## Data Availability

All data generated or analysed during this study are included in this published article.

## Abbreviations

SCI: Spinal cord injury
PDGF: Platelet-derived growth factor
PDGFRβ: Platelet-derived growth factor receptor beta
BMS: Basso Mouse Scale
ECM: Extracellular matrix
AD: Alzheimer’s disease
PD: Parkinson’s disease
ALS: Amyotrophic lateral sclerosis
PBS: Phosphate buffered saline
BSA: Bovine serum albumin
BrdU: Bromodeoxyuridine
PFA: Paraformaldehyde
HCl: Hydrochloric acid
5-HT: 5-Hydroxytryptamine
NF: Neurofilament
TGF-β: Transforming growth factor beta
epoB: Epothilone B
JAK: Janus kinase
PLCγ: Phospholipase C gamma
PI3K: Phosphoinositide 3-kinase
BBB: Blood–brain barrier
CSPGs: Chondroitin sulphate proteoglycans
